# Different trends in suicide rates among foreign residents in Japan and Japanese citizens during the COVID-19 pandemic

**DOI:** 10.1186/s12939-024-02234-z

**Published:** 2024-07-31

**Authors:** Yuta Taniguchi, Nanako Tamiya, Masao Iwagami, Kazumasa Yamagishi, Atsushi Miyawaki, Rie Masuda, Tomomi Kihara, Jun Komiyama, Hirokazu Tachikawa, Hideto Takahashi, Hiroyasu Iso

**Affiliations:** 1 Department of Health Services Research, Graduate School of Comprehensive Human Sciences, University of Tsukuba, 1-1-1 Tenno-dai, Tsukuba, Ibaraki 305-8575 Japan; 2 Institute for Global Health Policy Research, Bureau of International Health Cooperation, National Center for Global Health and Medicine, 1-21-1 Toyama, Shinjuku-ku, Tokyo, 162-8655 Japan; 3 Department of Health Services Research, Institute of Medicine, University of Tsukuba, 1-1-1 Tenno-dai, Tsukuba, Ibaraki 305-8575 Japan; 4 Health Services Research and Development Center, University of Tsukuba, 1-1-1 Tenno-dai, Tsukuba, Ibaraki 305-8575 Japan; 5 Faculty of Epidemiology and Population Health, London School of Hygiene and Tropical Medicine, Keppel Street, London, WC1E 7HT UK; 6 Department of Public Health Medicine, Institute of Medicine, University of Tsukuba, 1-1-1 Tenno-dai, Tsukuba, Ibaraki 305-8575 Japan; 7 Department of Public Health, Graduate School of Medicine, Juntendo University, 2-1-1 Hongo, Bunkyo-ku, Tokyo, 113-8421 Japan; 8 Department of Public Health, Graduate School of Medicine, The University of Tokyo, 7-3-1 Hongo, Bunkyo-ku, Tokyo, 113-0033 Japan; 9 Division of Nursing, Higashigaoka Faculty of Nursing, Tokyo Healthcare University, 2-5-1, Higashigaoka, Meguro-ku, Tokyo, 152-8558 Japan; 10 Department of Disaster and Community Psychiatry, Division of Clinical Medicine, Institute of Medicine, University of Tsukuba, 1-1-1 Tenno-dai, Tsukuba, Ibaraki 305-8575 Japan; 11 Faculty of Pharmaceutical Sciences, Teikyo Heisei University, 4-21-2 Nakano, Nakano-ku, Tokyo, 164-8530 Japan

**Keywords:** COVID-19, Suicide, Japan, Foreign residents, Japanese citizens

## Abstract

**Background:**

Suicide rates in Japan have increased during the COVID-19 pandemic, and foreign residents may be more vulnerable to mental stress during such crises. Therefore, we aimed to compare the trends in suicide rates during the COVID-19 pandemic between foreign residents and Japanese citizens.

**Methods:**

Vital statistics of Japan data from January 1, 2016 to December 31, 2021 were used to calculate quarterly sex-specific suicide rates for foreign residents and Japanese citizens. An event-study analysis was conducted to evaluate whether suicide rates during the COVID-19 pandemic increased compared to pre-pandemic estimates; foreign residents and Japanese citizens were compared using difference-in-difference-in-differences estimates.

**Results:**

Between 2016 and 2021, 1,431 foreign residents and 121,610 Japanese citizens died from suicide in Japan. Although the suicide rate for foreign residents was lower than that for Japanese citizens, Korean residents, who comprise approximately half of the foreign decedents, had largely higher suicide rates than Japanese citizens. The event-study analysis indicated that suicide rates increased among foreign residents for both men and women, and continued for men by the end of 2021. In Japanese citizens, after a decline in suicide rates in the second quarter of 2020, suicide rates increased both among men and women, and lasted for women until the fourth quarter of 2021. The difference-in-difference-in-differences analyses confirmed the initial decline in the second quarter of 2020 in suicide rate only in Japanese men and women, and the persistent increase through 2021 in foreign men.

**Conclusions:**

We found differential trends in suicide rates between foreign and Japanese men and women during the COVID-19 pandemic featuring a persistent increase in foreign men. Suicide prevention measures should be focused on these high-risk subpopulations.

**Supplementary Information:**

The online version contains supplementary material available at 10.1186/s12939-024-02234-z.

## Background

During the coronavirus disease 2019 (COVID-19) pandemic, Japan was one of the unique countries that showed an increase in suicide rates [1–9], with suicide rates before the pandemic ranking top among G7 countries and fifth among the 38 OECD countries [10]. Earlier studies reported that the increased suicide rates during the COVID-19 pandemic were especially prominent among women [2, 3, 5–7, 9], the youth [1, 3, 5], housewives [3], urban residents [5], and persons living alone [5]. However, to the best of our knowledge, no studies have compared the situations of suicide between foreign residents and native residents. In general, foreign residents are assumed to be vulnerable populations, owing to the inequity of social, economic, and medical care conditions [11–13]. Indeed, since the COVID-19 pandemic began in 2020, foreign residents have been reported to experience higher morbidity and mortality due to the virus [14], higher levels of distress, [15] and limited access to healthcare services, including COVID-19 vaccination [16, 17]. Considering the vulnerability of foreign residents during the COVID-19 pandemic, it is conceivable that foreign residents in Japan may have also experienced an increase in the suicide rates during the pandemic, even larger and more persistent than that experienced by Japanese citizens.

Despite increasing concern regarding the mental health of foreign residents, previous studies in Japan are limited, because they did not distinguish foreign residents and Japanese citizens when assessing the trends in suicide rates during the pandemic. Therefore, in this study, we aimed to address the following research questions; (1) Did suicide rates among foreign residents in Japan increase during the COVID-19 pandemic? and (2) Did the change in suicide rates vary between foreign residents and Japanese citizens?

## Methods

### Data source and study population

We acquired the vital statistics death data of Japan from January 1, 2016 to December 31, 2021 from the Ministry of Health, Labour, and Welfare, under the Statistics Act, Article 33. Vital statistics of Japan records every death, including date and cause, as well as the deceased’s age, sex, and nationality [18]. The validity of the vital statistics data is rigorously maintained under the supervision of the Ministry of Health, Labour, and Welfare.

We identified individuals who died by suicide, using the tenth revision of the International Statistical Classification of Diseases (ICD-10) codes X60–X84. The individuals with missing data for age, sex, or nationality were excluded from the analysis.

### Outcome measure

The outcome measure was the sex-specific crude suicide rate in each quarter (January to March, April to June, July to September, and October to December) from 2016 to 2021. We calculated the quarterly suicide rate for both foreign residents and Japanese citizens by dividing the number of suicides by the population in each quarter. For Japanese citizens, we extracted the data on populations from the monthly population estimates [19], summed up the monthly numbers and took the average for each quarter. For foreign residents, as the population of foreign residents is reported only twice a year (June and December) by the Statistics on Foreign National Residents in Japan [20], we substituted the population in June for the second and third quarter, and that in December for the fourth quarter and the first quarter of the following year. Population of foreign residents included any foreign national who stayed in Japan during the period of measurement.

### Statistical analysis

First, we compared the basic characteristics of foreign residents and Japanese citizens who died from suicide from 2016 to 2021. Second, we calculated and plotted the sex-specific suicide rate for each quarter of the observed years for foreign residents and Japanese citizens separately. We also stratified the study participants into three age groups (< 40, 40–59, or ≥ 60 years old). We then calculated the sex-specific suicide rates for Koreans, who were reported as having higher suicide rates than other foreign residents in Japan and Japanese citizens before the COVID-19 pandemic [21, 22]. Third, to estimate the association between the pandemic suicide rates by sex and nationality (foreign or Japanese), we used an event-study difference-in-differences analysis (hereafter, event-study analysis) and compared the change in suicide rates in the event period (first quarter of 2019 to fourth quarter of 2021; the event time was the “beginning” of the pandemic at the end of first quarter of 2020) with the three years earlier as the control period (first quarter of 2016 to fourth quarter of 2018). The event-study analysis allowed us to observe a longitudinal trend of suicide rates at multiple time points after the COVID-19 pandemic by comparing the corresponding pre-pandemic period. The end of the first quarter of 2020 (April 2020) was defined as the beginning of the COVID-19 pandemic, corresponding to when the Japanese government declared a state of emergency [23].

We denoted the period from the first quarter (Q1) of 2019 to the fourth quarter (Q4) of 2021 as the “exposed group” and Q1 of 2016 to Q4 of 2018 as the “control group.” In the exposed group, the pandemic time frames were compared with pre-pandemic control time frames in terms of changes in suicide rates between the average of five quarters before the pandemic (Q1–Q4 of 2019 and Q1 of 2020) and each of seven subsequent quarters (Q2–Q4 of 2020 and Q1–Q4 of 2021). These were then compared with the changes in the corresponding quarters from the control group. We estimated a linear regression model with heteroskedasticity-robust standard errors (see Supplementary Method for more details on the statistical analyses). Fourth, to examine whether the event-study estimates at each quarter varied between the foreign residents and Japanese citizens, we calculated the difference-in-difference-in-differences estimates (triple difference estimator), which is measured as the difference between two difference-in-differences or event-study estimates [24]. We also conducted the stratified analyses for Koreans and other foreign nationals.

Analyses were performed using Stata 17 (StataCorp, TX, USA) and Microsoft Excel for Mac 16.67 (Microsoft, WA, USA).

## Results

We identified 123,041 individuals (1,431 foreign residents and 121,610 Japanese citizens) who died by suicide in Japan between 2016 and 2021, after excluding 313 persons with missing variables of age, sex, or nationality. As shown in Table 1, the proportion of women among foreign residents and Japanese citizens was 37.5% and 31.2%, respectively. The median age was 48 (interquartile range [IQR], 31–63) for foreign men, 50 (35–63) for foreign women, 52 (38–68) for Japanese men, and 55 (39–72) for Japanese women. The annual number of deaths were between 207 and 265 for foreign residents and around 20,000 for Japanese citizens. Koreans constituted half of the foreign residents for both sexes, and as shown in Supplementary Table 1, Koreans were more likely to be older and unemployed, compared with other foreign residents.

**Table 1:** Baseline characteristics of individuals who died by suicide in Japan between 2016–2021 (*n* = 123,041)

	Foreign residents (*n* = 1431)		Japanese citizens (*n* = 121,610)	
	Men, *n* (%) 895 (62.5)	Women, *n* (%) 536 (37.5)	Men, *n* (%) 83,699 (68.8)	Women, *n* (%) 37,911 (31.2)
Age, median (IQR), year	48 (31–63)	50 (35–63)	52 (38–68)	55 (39–72)
Nationality, *n* (%)				
Korea	451 (50.4)	274 (51.1)	–	–
China	114 (12.7)	129 (24.1)	–	–
Brazil	79 (8.8)	15 (2.8)	–	–
Philippines	24 (2.7)	42 (7.8)	–	–
United States	33 (3.7)	5 (0.9)	–	–
Thailand	10 (1.1)	13 (2.4)	–	–
Peru	6 (0.7)	1 (0.2)	–	–
United Kingdom	4 (0.5)	2 (0.4)	–	–
Others	174 (19.4)	55 (10.3)	–	–
Occupation, *n* (%)				
Agriculture	7 (0.8)	4 (0.8)	3646 (4.4)	1422 (3.8)
Self-employed	65 (7.3)	42 (7.8)	6094 (7.3)	2224 (5.9)
Employee	178 (19.9)	94 (17.5)	20,961 (25.0)	8383 (22.1)
Other	103 (11.5)	76 (14.2)	11,316 (13.5)	4622 (12.2)
Not working	369 (41.2)	218 (40.7)	32,948 (39.4)	17,515 (46.2)
Unknown	173 (19.3)	102 (19.0)	8734 (10.4)	3745 (9.9)
Marital status *n* (%)				
Married	288 (32.2)	198 (36.9)	31,980 (38.2)	15,142 (39.9)
Not married	368 (41.1)	169 (31.5)	33,470 (40.0)	10,460 (27.6)
Bereaved	31 (3.5)	57 (10.6)	5052 (6.0)	6780 (17.9)
Divorced	137 (15.3)	93 (17.4)	12,959 (15.5)	5475 (14.4)
Unknown	71 (7.9)	19 (3.5)	238 (0.3)	54 (0.1)
No of deaths, *n* (%)				
2016	146 (16.3)	81 (15.1)	14,663 (17.5)	6382 (16.8)
2017	133 (14.9)	74 (13.8)	14,354 (17.2)	6134 (16.2)
2018	147 (16.4)	98 (18.3)	13,865 (16.6)	6190 (16.3)
2019	146 (16.3)	78 (14.6)	13,711 (16.4)	5764 (15.2)
2020	156 (17.4)	107 (20.0)	13,631 (16.3)	6662 (17.6)
2021	167 (18.7)	98 (18.3)	13,475 (16.1)	6779 (17.9)

Abbreviations: IQR, interquartile range

Occupation refers to the occupation of the highest earner in the household.

**Fig. 1:**
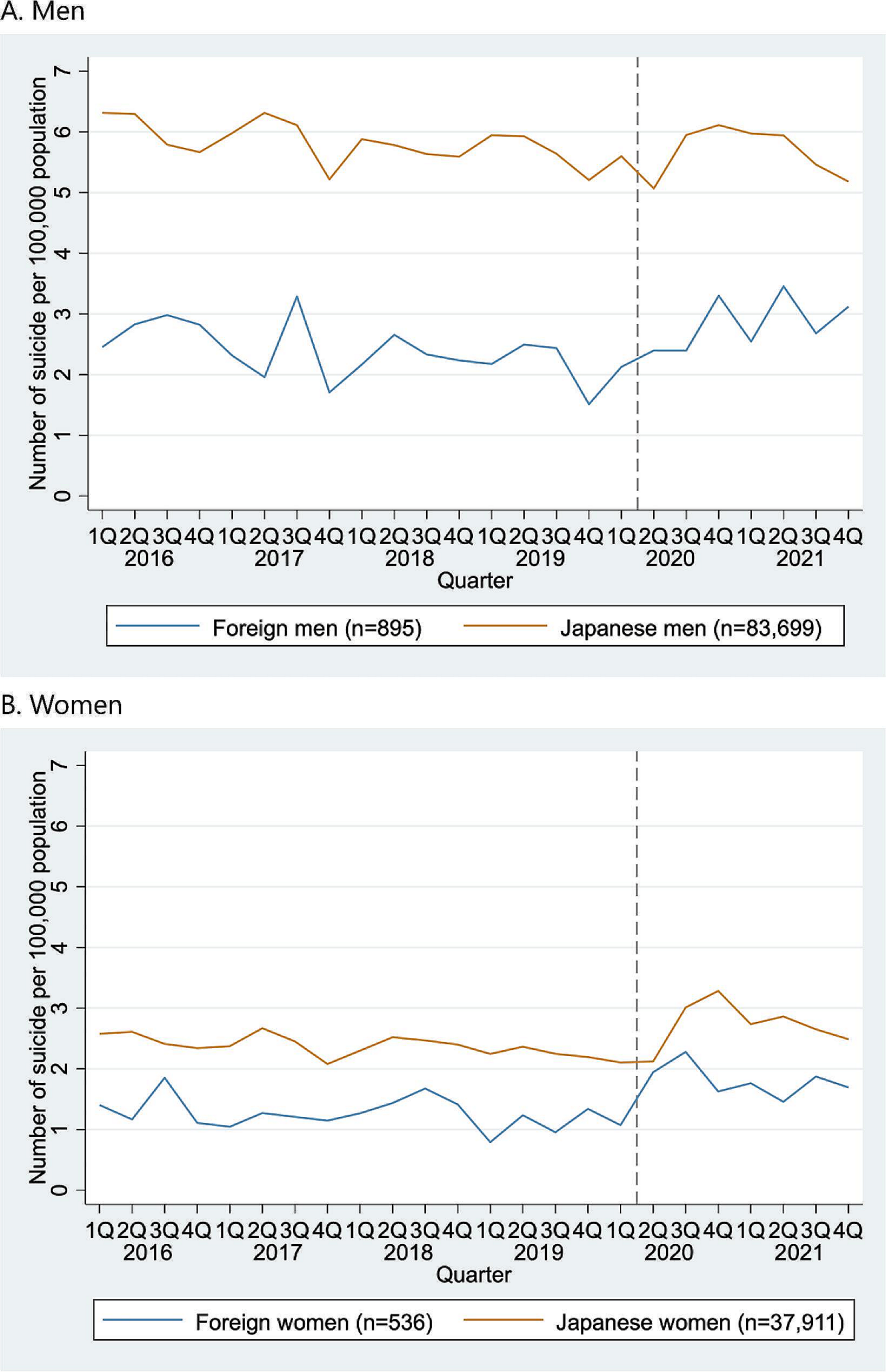
Quarterly suicide rates among foreign residents in Japan and Japanese citizens from 2016–2021. Abbreviations: Q, Quarter Note: The gray dashed lines represent the beginning of the COVID-19 pandemic in Japan (April 2020)

**Fig. 2:**
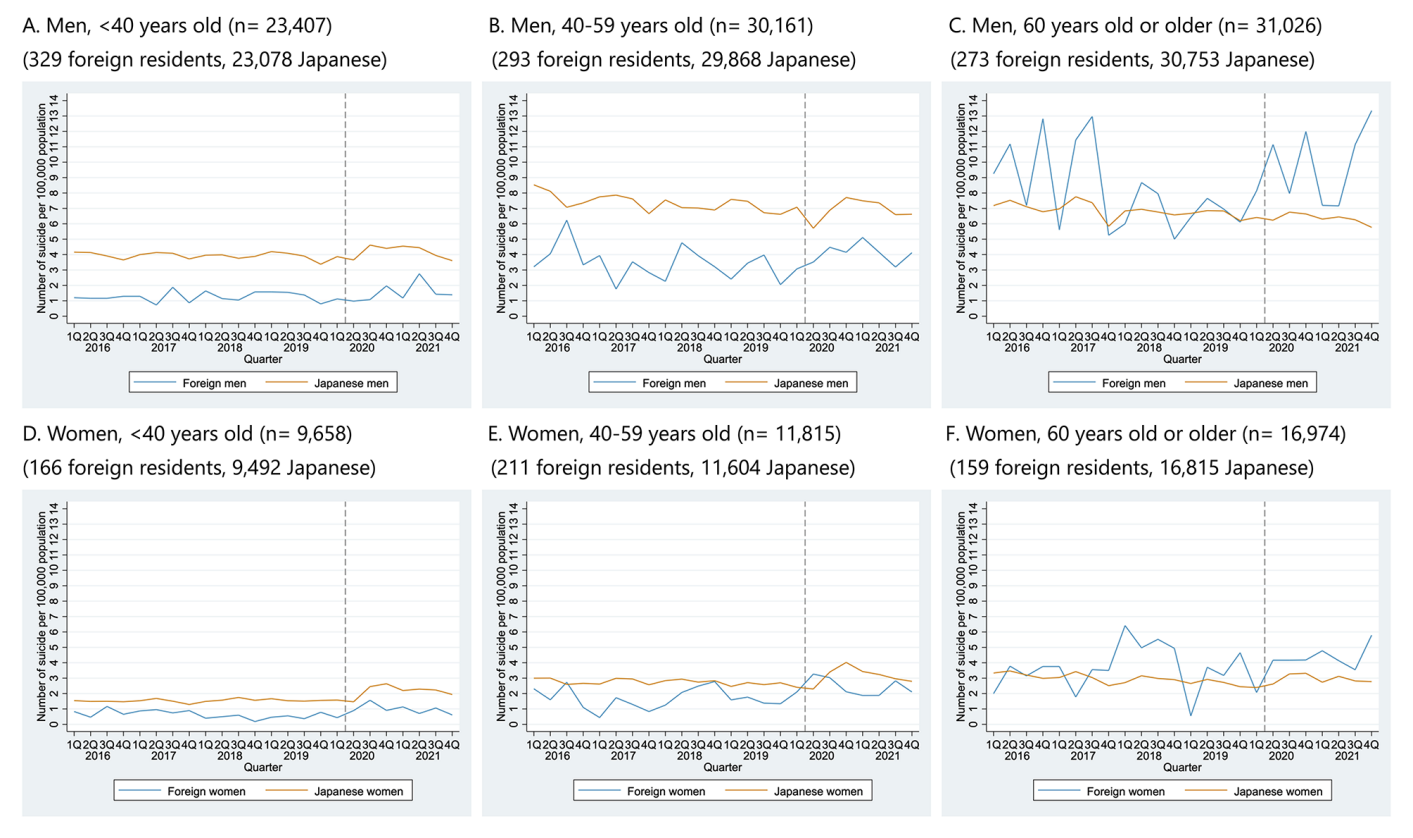
Quarterly suicide rates among foreign residents in Japan and Japanese citizens from 2016–2021, by age group. Abbreviations: Q, Quarter Note: The gray dashed lines represent the beginning of the COVID-19 pandemic in Japan (April 2020)

As shown in Fig. 1, for both men and women, the quarterly suicide rates were higher among Japanese citizens than foreign residents throughout the study period; this is partly because of the higher mean age among Japanese citizens, compared to foreign residents. When stratified by age group, such differences were evident at ages of < 40 and 40–59 years (Fig. 2). When we stratified foreign residents into Koreans and other nationalities, Korean people experienced higher suicide rates than other foreign residents and Japanese citizens, for both men and women throughout the study period (Supplementary Fig. 1), particularly among men aged ≥ 60 (Supplementary Fig. 2).

**Fig. 3:**
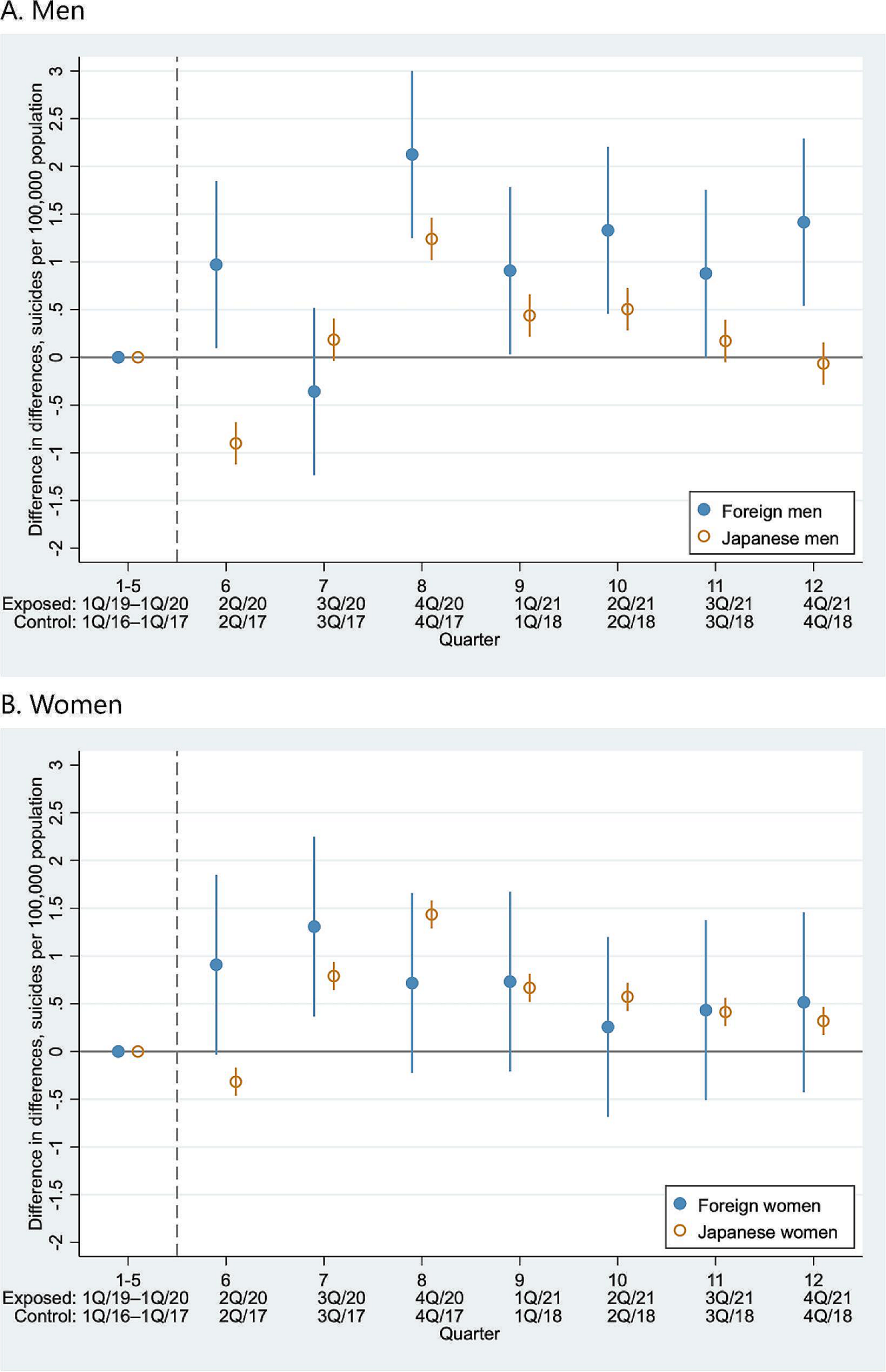
Event-study estimates of suicide rates among foreign residents in Japan and Japanese citizens from 2019–2021 vs. 2016–2018. Abbreviations: Q, Quarter. Note: The bars indicate 95% confidence intervals, and the gray dashed lines indicate the beginning of the COVID-19 pandemic (April 2020)

Figure 3 presents the event-study analysis estimates of the sex-specific suicide rates among foreign residents and Japanese citizens. In the exposed group (2019–2021), we observed increased suicide rates among foreign men in the second and fourth quarters of 2020 and all quarters of 2021, and among foreign women in the third quarter of 2020, compared to the control group (2016–2018). For Japanese citizens, after a decline in suicide rates in the second quarter of 2020 for both men and women, suicide rates increased among men from the fourth quarter of 2020 to the second quarter of 2021, and among women from the third quarter of 2020 to the fourth quarter of 2021.

As shown in Table 2, the difference-in-difference-in-differences analyses showed a greater increase in suicide rates among foreign men, compared with Japanese men, in the second and fourth quarters of 2020, as well as from the second to the fourth quarters of 2021. The increase in the suicide rate was larger for foreign women in the second quarter of 2020, and for Japanese women in the fourth quarter of 2020, followed by no difference through the end of 2021.

When we stratified foreign residents into Korean nationals and other nationalities, the event-study analysis for Korean men showed that suicide rates increased in the fourth quarter of 2020, and from the third to the fourth quarters in 2021; however, we observed no differences among Korean women (Supplementary Fig. 3). As shown in Supplementary Table 2, the difference-in-difference-in-differences analyses confirmed the increase in suicide rates in late 2021 among Korean men, compared with Japanese men, until the end of 2021.

**Table 2:** Difference-in-difference-in-differences (DDD) estimates of the effects of the COVID-19 pandemic on suicide rates in foreign residents and Japanese citizens

	Men			Women		
	Japanese citizens	Foreign residents		Japanese citizens	Foreign residents	
	DD estimates (95% CI)	DD estimates (95% CI)	DDD estimates, foreign residents vs. Japanese (95% CI)	DD estimates (95% CI)	DD estimates (95% CI)	DDD estimates, foreign residents vs. Japanese (95% CI)
Q2, 2020	-0.90*** (-1.12, -0.68)	0.97* (0.096, 1.85)	1.87*** (1.20, 2.54)	-0.32** (-0.46, -0.17)	0.91 (-0.03, 1.85)	1.23** (0.51, 1.94)
Q3, 2020	0.18 (-0.04, 0.41)	-0.36 (-1.23, 0.52)	-0.54 (-1.22, 0.13)	0.79*** (0.64, 0.94)	1.31* (0.37, 2.25)	0.52 (-0.19, 1.23)
Q4, 2020	1.24*** (1.02, 1.46)	2.13** (1.25, 3.00)	0.88* (0.21, 1.56)	1.44*** (1.29, 1.58)	0.72 (-0.22, 1.66)	-0.72* (-1.43, -0.01)
Q1, 2021	0.44** (0.22, 0.66)	0.91* (0.033, 1.78)	0.47 (-0.20, 1.14)	0.67*** (0.52, 0.81)	0.73 (-0.21, 1.67)	0.06 (-0.65, 0.78)
Q2, 2021	0.50* (0.28, 0.73)	1.33* (0.46, 2.21)	0.83* (0.15, 1.50)	0.57*** (0.43, 0.72)	0.26 (-0.69, 1.20)	-0.32 (-1.03, 0.39)
Q3, 2021	0.17 (-0.05, 0.39)	0.88* (0.0035, 1.75)	0.71* (0.03, 1.38)	0.41** (0.27, 0.56)	0.43 (-0.51, 1.38)	0.02 (-0.69, 0.73)
Q4, 2021	-0.07 (-0.29, 0.16)	1.42* (0.54, 2.29)	1.48*** (0.81, 2.15)	0.32** (0.17, 0.47)	0.52 (-0.43, 1.46)	0.20 (-0.51, 0.91)

Abbreviations: DD, difference-in-differences; DDD, difference-in-difference-in-differences; CI, confidence interval; Q, Quarter

* *P* < 0.05, ** *P* < 0.01, ***P < 0.001

## Discussion

Using the vital statistics data of Japan for the period 2016–2021, we found differential sex-specific trends for quarterly suicide rates during the COVID-19 pandemic from April 2020 to December 2021 between foreign residents and Japanese citizens. Suicide rates increased among foreign men in the second and fourth quarters of 2020 and all quarters of 2021, and among foreign women in the third quarter of 2020. For Japanese citizens, after an initial decline in the second quarter of 2020, suicide rates increased among men from the fourth quarter of 2020 to the second quarter of 2021, and among women from the third quarter of 2020 to the fourth quarter of 2021. The difference-in-difference-in-differences analyses confirmed the initial decline in suicide rates only among Japanese men and women, and the persistent increase through 2021 among foreign men. To the best of our knowledge, this is the first study to evaluate the sex- and nationality-specific trends in suicide rates during the COVID-19 pandemic.

The lower suicide rates among foreign residents compared to Japanese citizens before and during the COVID-19 pandemic were partly due to a younger age; when stratified by age group, such trends were observed for ages < 40 and 40–59 years, but not for ages ≥ 60 years. Additionally, Korean residents experienced higher suicide rates than other foreign nationals or Japanese citizens, particularly men aged ≥ 40 and women aged < 60 years. Previous studies reported higher suicide rates in Korean residents, compared to Japanese citizens, prior to the COVID-19 pandemic [21, 22], and our results suggest that this phenomenon continued during the pandemic.

Previous studies have reported the initial decline in suicide rates during the COVID-19 pandemic in Japan [2–5, 7]. It was suggested that this may be attributable to increased feelings of social connectedness in a time of crisis (also called the “pulling-together effect”) [2–4], reduced working hours, and subsidies from the government [3]. On the contrary, we did not observe such decline among foreign residents, which may be due to the less availability of aforementioned protective factors. Some foreign residents could not return to their home countries due to the cancellation of flights [25], and may have faced greater difficulties than Japanese citizens.

It is noteworthy that the increase in the suicide rates continued for foreign men, but not for Japanese men, until the end of 2021. According to the population census of October 2020, foreign men had a higher unemployment rate (4.9%) than Japanese men (4.3%), and they may have been more likely to experience economic difficulties [26]. Korean men had a particularly high unemployment rate (6.9%), and because around a quarter of them worked as sales or service workers [26], they may have experienced financial crisis during the pandemic, which could had made them particularly vulnerable.

Historically, the suicide rate in Japan increased rapidly in the late 1990s, and the Japanese government worked on suicide prevention policies, ratifying the Basic Act on Suicide Prevention in 2006 [27]. Although the suicide rate in Japan decreased from 2011, it resurged during the COVID-19 pandemic. Thus, the reinforcement of suicide prevention with a focus on socially disadvantaged populations is important. In this context, our study illuminated the possible importance of extending suicide prevention for foreign residents in Japan, as well as Japanese citizens. For example, the Ministry of Health, Labour, and Welfare of Japan published a website in Japanese that provides a wide variety of information on available mental health services, such as telephone consultation services [28]. However, resources in other languages are still limited, despite the language barrier being reported as the major issue for mental well-being among international migrants in Japan [29]. Thus, the enhancement of multilingual information and assistance through governments and non-governmental organizations is highly recommended for socially disadvantaged populations.

The study has several strengths. First, to our knowledge, this is the first study to investigate trends in suicide rates during the COVID-19 pandemic, focusing on differences by nationalities. Besides, this study provided the most up-to-date information up to the end of 2021, using the most recent definitive statistics of the Japanese vital statistics available.

This study also has several limitations that must be noted. First, the residential status or employment conditions could vary largely among foreign residents with undocumented, short-term, or long-term ones, and suicide risk may differ accordingly. However, we could not acquire this information. Yet, our study showed higher suicide rates among Koreans, the majority of whom were long-term residents [20]. Second, because we did not have information on the reasons for suicide, which could be multifactorial, we could not determine if the increases in suicide rates were caused directly or indirectly by the COVID-19 pandemic. Still, our findings highlight the need for the promotion of mental health services to prevent suicide for both Japanese and foreign residents. Third, although financial problems and a history of mental disorders were reported as risk factors for suicide [30], we could not evaluate the differences in economic conditions or health status between Japanese citizens and foreign residents. Differences in economic conditions and health status may partly explain the different trends in suicide rates by nationality. Future studies using the linked data of census, medical records, and vital statistics would be necessary. Lastly, since the Japanese government records the census of foreign residents only bi-annually, in June and December, we may have overlooked the changes in the populations of foreign residents at the quarterly level; however, it is unknown whether the difference in census procedures would affect the suicide rates. As a sensitivity analysis, we calculated the quarterly suicide rate for Japanese citizens in the same way as for foreign residents. That is, as the denominator, we used the population in June for the second and third quarters and that in December for the fourth quarter and the first quarter of the following year. The results were almost identical.

## Conclusions

In conclusion, during the COVID-19 pandemic, we observed an increase in suicide rates among both foreign residents and Japanese citizens but found an initial decline only among Japanese citizens. Our findings imply a need for tailored mental health support as well as reasonable job opportunities for vulnerable populations, especially during pandemics with substantial impact on socioeconomic conditions.

### Electronic supplementary material

Below is the link to the electronic supplementary material.

Supplementary Material 1: Supplementary Method 1. Event-study difference-in-differences analysis. Supplementary Table 1. Baseline characteristics of individuals who died by suicide in Japan between 2016 and 2021. Supplementary Fig. 1. Quarterly suicide rates among Koreans and other foreign nationals in Japan and Japanese citizens from 2016 to 2021. Supplementary Fig. 2. Quarterly suicide rates among Koreans and other foreign nationals in Japan and Japanese citizens from 2016 to 2021, by age group. Supplementary Fig. 3. Event-study estimates of suicide rates among Koreans and other foreign nationals in Japan and Japanese citizens from 2019 to 2021 vs. 2016–2018. Supplementary Table 2. Difference-in-difference-in-differences (DDD) estimates of the effects of the COVID-19 pandemic on suicide rates in Korean residents and Japanese citizens.

## Data Availability

Data cannot be shared because the Japanese Ministry of Health, Labour and Welfare owns the original data and only approved its secondary use for the current study. Researchers who meet the criteria may apply directly to the Ministry for permission to use the deidentified participant data.

## Abbreviations

ICD-10: International Statistical Classification of Diseases, Tenth Revision
IQR: Interquartile range
Q: Quarter
DD: Difference-in-differences
DDD: Difference-in-difference-in-differences
CI: Confidence interval
