# Supplementary material for: Different trends in suicide rates among foreign residents in Japan and Japanese citizens during the COVID-19 pandemic
Source: Int J Equity Health. 2024 Jul 31;23:150. doi: 10.1186/s12939-024-02234-z (PMC11290109; doi:10.1186/s12939-024-02234-z)
Supplement: Supplementary file 1 — Supplementary Material 1: Supplementary Method 1. Event-study difference-in-differences analysis. Supplementary Table 1. Baseline characteristics of individuals who died by suicide in Japan between 2016 and 2021. Supplementary Fig. 1. Quarterly suicide rates among Koreans and other foreign nationals in Japan and Japanese citizens from 2016 to 2021. Supplementary Fig. 2. Quarterly suicide rates among Koreans and other foreign nationals in Japan and Japanese citizens from 2016 to 2021, by age group. Supplementary Fig. 3. Event-study estimates of suicide rates among Koreans and other foreign nationals in Japan and Japanese citizens from 2019 to 2021 vs. 2016–2018. Supplementary Table 2. Difference-in-difference-in-differences (DDD) estimates of the effects of the COVID-19 pandemic on suicide rates in Korean residents and Japanese citizens. [file 12939_2024_2234_MOESM1_ESM.pdf]

Supplementary Material

Different trends in suicide rates among foreign residents in Japan and Japanese citizens during the COVID-19 pandemic

Supplementary Method 1. Event-study difference-in-differences analysis.

Supplementary Table 1. Baseline characteristics of individuals who died by suicide in Japan between 2016–2021.

Supplementary Figure 1. Quarterly suicide rates among Koreans and other foreign nationals in Japan and Japanese citizens from 2016–2021.

Supplementary Figure 2. Quarterly suicide rates among Koreans and other foreign nationals in Japan and Japanese citizens from 2016–2021, by age group.

Supplementary Figure 3. Event-study estimates of suicide rates among Koreans and other foreign nationals in Japan and Japanese citizens from 2019–2021 vs 2016–2018.

Supplementary Table 2. Difference-in-difference-in-differences (DDD) estimates of the effects of the COVID-19 pandemic on suicide rates in Korean residents and Japanese citizens.

Supplementary Method 1. Event-study difference-in-differences analysis.

For the event-study difference-in-differences analysis, we estimated the following model:

$$Outcome_q = \alpha * Exposed_t + \beta_q + \sum_{q=6}^{12} \gamma_q * Exposed_t \times 1(Q = q) + \varepsilon \quad (1)$$

where $Outcome_q$ is the suicide rate at quarter q. Quarter q represents the qth quarter, counting from the beginning of 2019 (for the exposed group) and 2016 (for the control group), which takes a value from 1 to 12. Given the pandemic (= "exposure") began in April 2020, we considered the average of the first to fifth quarter (q=1–5) as the reference. Variable $Exposed_t$ takes a value of 1 for 2019–2021 (exposed group) and 0 for 2016–2018 (control group), and $1(Q = q)$ is an indicator variable that takes a value of 1 for each quarter q from 6 to 12, and zero otherwise. β_q is the fixed effect for each quarter to account for seasonality. ε is the error term. As such, $Exposed_t \times 1(Q = q)$ represents each quarter of the pandemic timeframe. The estimates of γ_q for each quarter were plotted to evaluate the dynamics of the effect of the pandemic on suicide rates in the "post-exposure" period (second quarter of 2020 to the fourth quarter of 2021 vs. the second quarter of 2017 to fourth quarter of 2018). The coefficient was considered statistically significant if the 95% confidence interval did not overlap with zero.

Supplementary Table 1. Baseline characteristics of individuals who died by suicide in Japan between 2016–2021 (n = 123,041).

	Koreans (n = 725)		Foreign residents (other than Koreans) (n = 706)		Japanese citizens (n = 121,610)	
	Men, n (%)	Women, n (%)	Men, n (%)	Women, n (%)	Men, n (%)	Women, n (%)
	451 (62.2)	274 (37.8)	444 (62.9)	262 (37.1)	83,699 (68.8)	37,911 (31.2)
Age, median (IQR), year	58 (45–70)	58 (42–68)	36 (26–51)	44 (29–55)	52 (38–68)	55 (39–72)
Occupation, n (%)						
Agriculture	1 (0.2)	1 (0.4)	6 (1.4)	3 (1.2)	3,646 (4.4)	1,422 (3.8)
Self-employed	41 (9.1)	22 (8.0)	24 (5.4)	20 (7.6)	6,094 (7.3)	2,224 (5.9)
Employee	67 (14.9)	37 (13.5)	111 (25.0)	57 (21.8)	20,961 (25.0)	8,383 (22.1)
Other	44 (9.8)	27 (9.9)	59 (13.3)	49 (18.7)	11,316 (13.5)	4,622 (12.2)
Not working	213 (47.2)	123 (44.9)	156 (35.1)	95 (36.3)	32,948 (39.4)	17,515 (46.2)
Unknown	85 (18.9)	64 (23.4)	88 (19.8)	38 (14.5)	8,734 (10.4)	3,745 (9.9)
Marital status, n (%)						
Married	140 (31.0)	86 (31.4)	148 (33.3)	112 (42.8)	31,980 (38.2)	15,142 (39.9)
Not married	162 (35.9)	85 (31.0)	206 (46.4)	84 (32.1)	33,470 (40.0)	10,460 (27.6)
Bereaved	23 (5.1)	40 (14.6)	8 (1.8)	17 (6.5)	5,052 (6.0)	6,780 (17.9)
Divorced	89 (19.7)	51 (18.6)	48 (10.8)	42 (16.0)	12,959 (15.5)	5,475 (14.4)
Unknown	37 (8.2)	12 (4.4)	34 (7.7)	7 (2.7)	238 (0.3)	54 (0.1)
No of deaths, n (%)						
2016	77 (17.1)	43 (15.7)	69 (15.5)	38 (14.5)	14,663 (17.5)	6,382 (16.8)
2017	78 (17.3)	41 (15.0)	55 (12.4)	33 (12.6)	14,354 (17.2)	6,134 (16.2)
2018	77 (17.1)	55 (20.1)	70 (15.8)	43 (16.4)	13,865 (16.6)	6,190 (16.3)
2019	68 (15.1)	38 (13.9)	78 (17.6)	40 (15.3)	13,711 (16.4)	5,764 (15.2)
2020	78 (17.3)	51 (18.6)	78 (17.6)	56 (21.4)	13,631 (16.3)	6,662 (17.6)
2021	73 (16.2)	46 (16.8)	94 (21.2)	52 (19.9)	13,475 (16.1)	6,779 (17.9)

Supplementary Figure 1. Quarterly suicide rates among Koreans and other foreign nationals in Japan and Japanese citizens from 2016–2021.

A. Men

(451 Korean men, 444 foreign men (other than Koreans), 83,699 Japanese men)

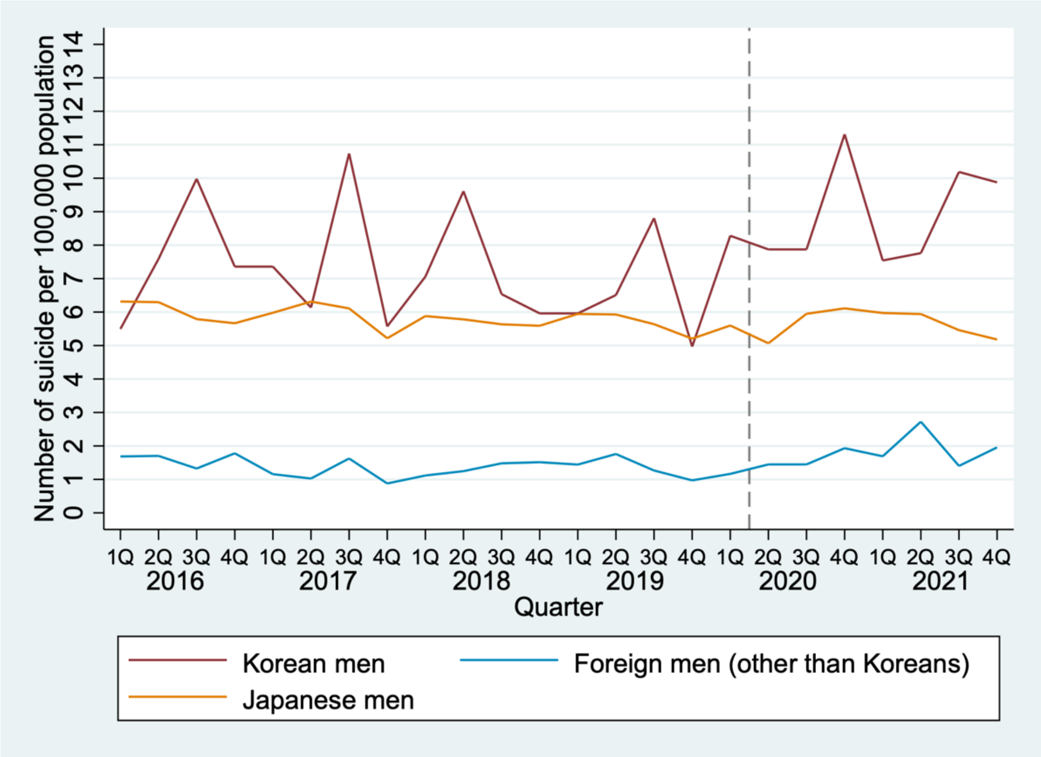

B. Women

(274 Korean women, 262 foreign women (other than Koreans), 37,911 Japanese women)

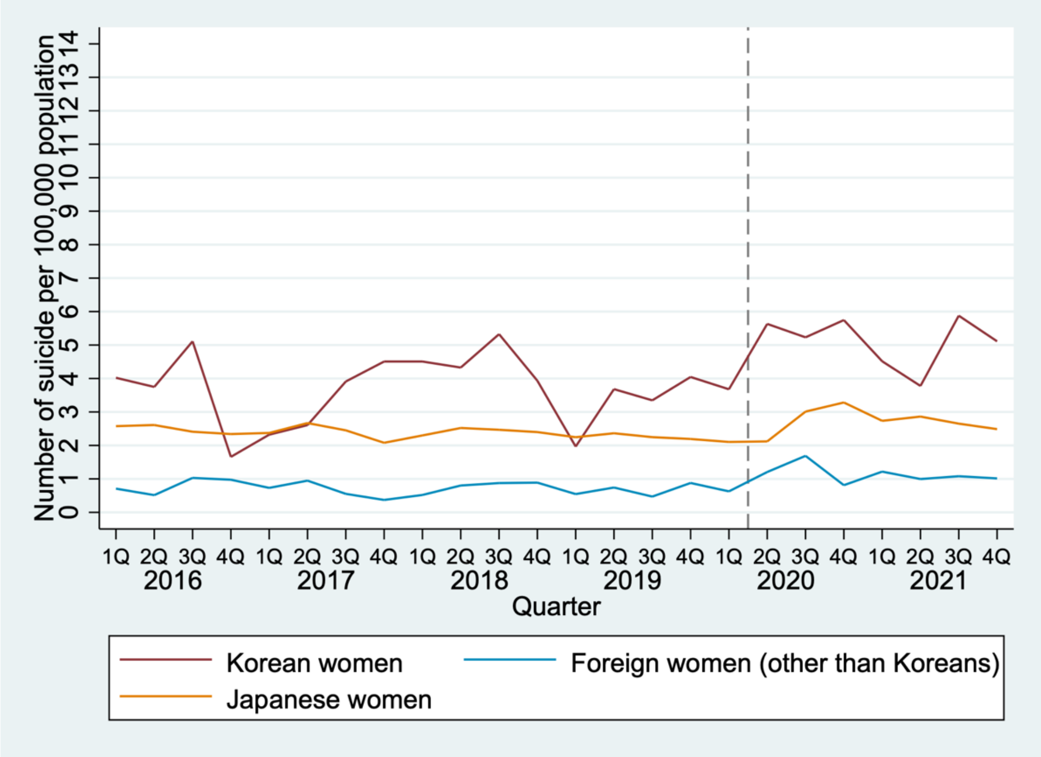

Note: The gray dashed lines represent the beginning of the COVID-19 pandemic in Japan (April 2020).

Supplementary Figure 2. Quarterly suicide rates among Koreans and other foreign nationals in Japan and Japanese citizens from 2016–2021, by age group.

A. Men, <40 years old (n= 23,407)

(80 Koreans, 249 other foreign residents, 23,078 Japanese)

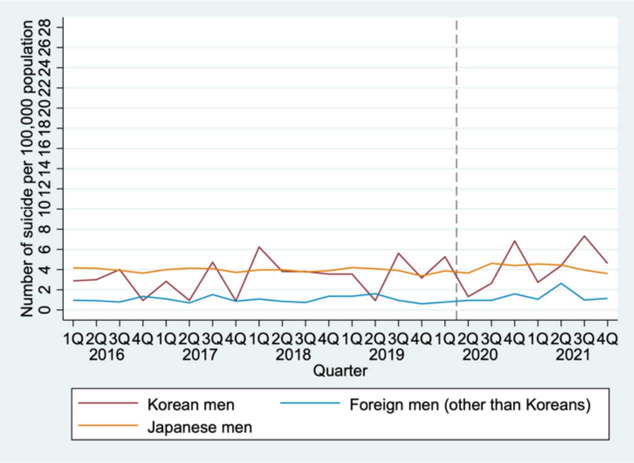

B. Men, 40-59 years old (n= 30,161)

(161 Koreans, 132 other foreign residents, 29,868 Japanese)

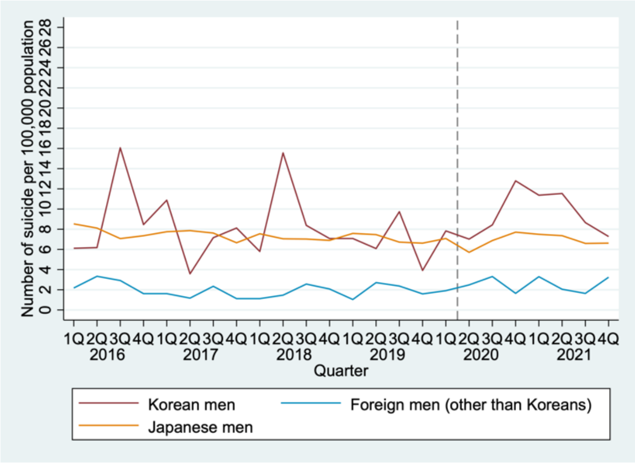

C. Men, 60 years old or older (n= 31,026)

(210 Koreans, 63 other foreign residents, 30,753 Japanese)

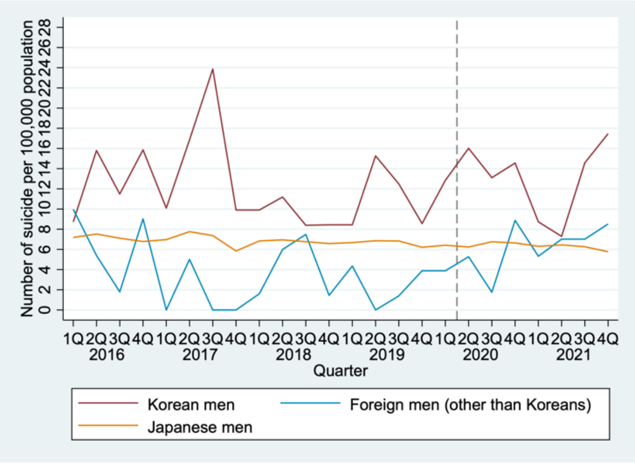

D. Women, <40 years old (n= 9,658)

(58 Koreans, 108 other foreign residents, 9,492 Japanese)

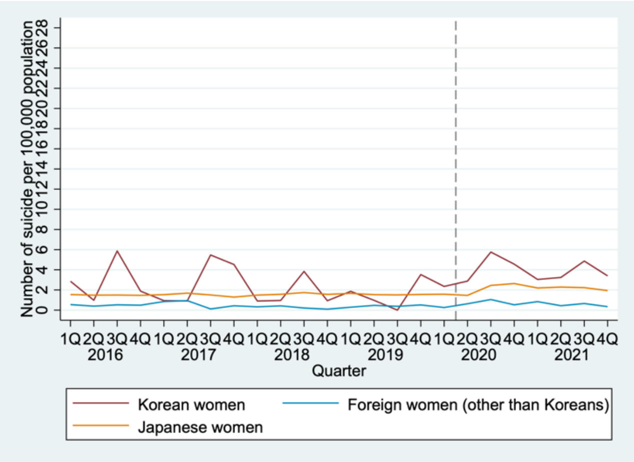

E. Women, 40-59 years old (n= 11,815)

(100 Koreans, 111 other foreign residents, 11,604 Japanese)

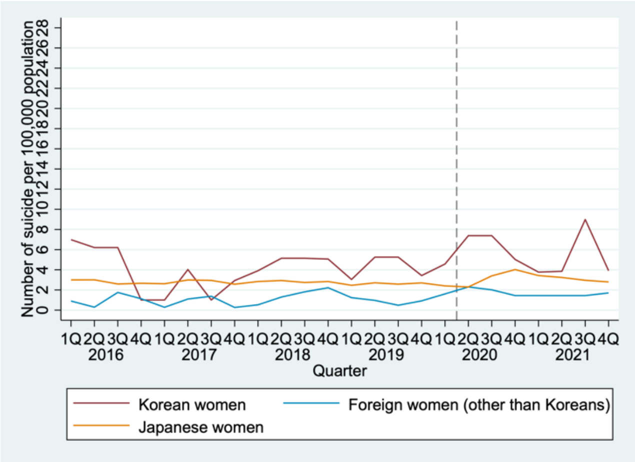

F. Women, 60 years old or older (n= 16,974)

(116 Koreans, 43 other foreign residents, 16,815 Japanese)

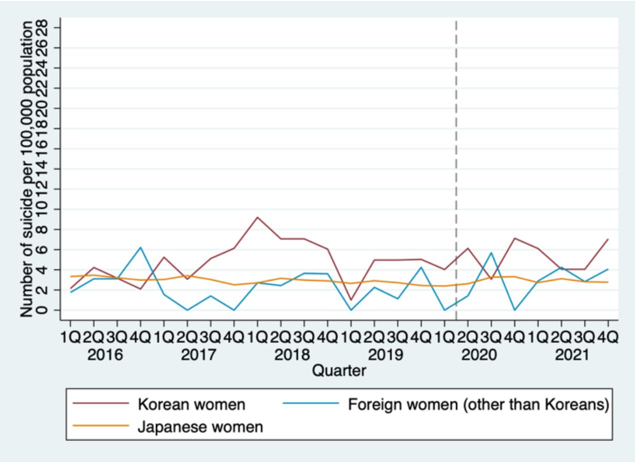

Note: The gray dashed lines represent the beginning of the COVID-19 pandemic in Japan (April 2020).

Supplementary Figure 3. Event-study estimates of suicide rates among Koreans and other foreign nationals in Japan and Japanese citizens from 2019–2021 vs 2016–2018.

A. Men

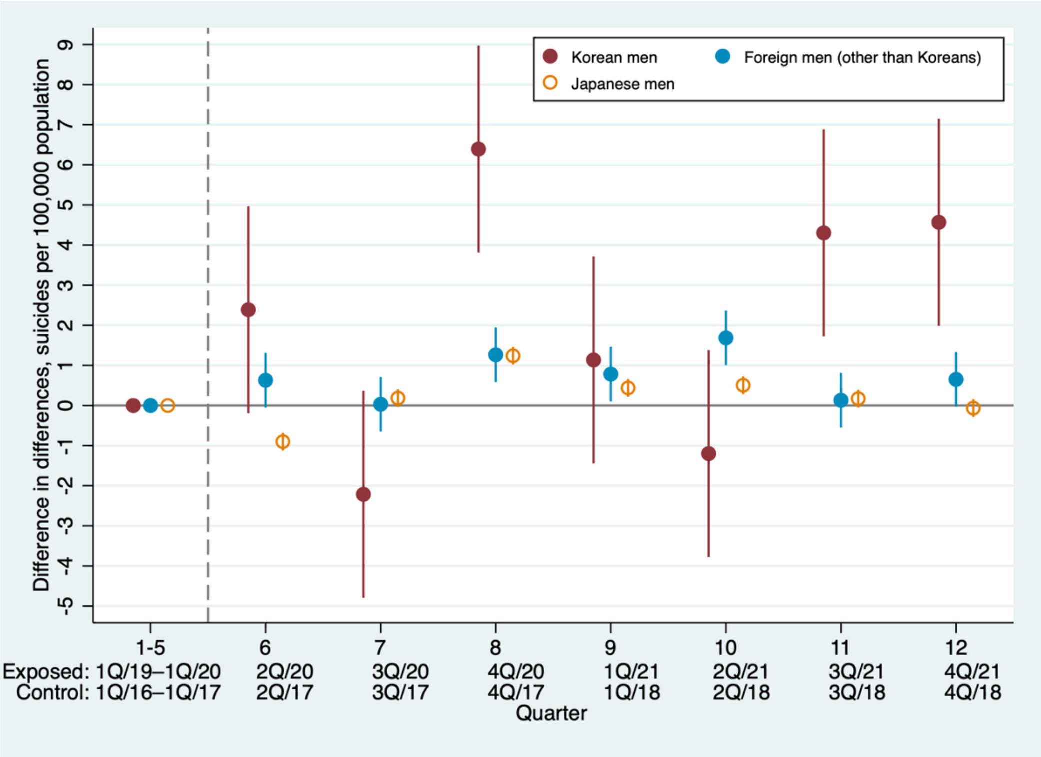

B. Women

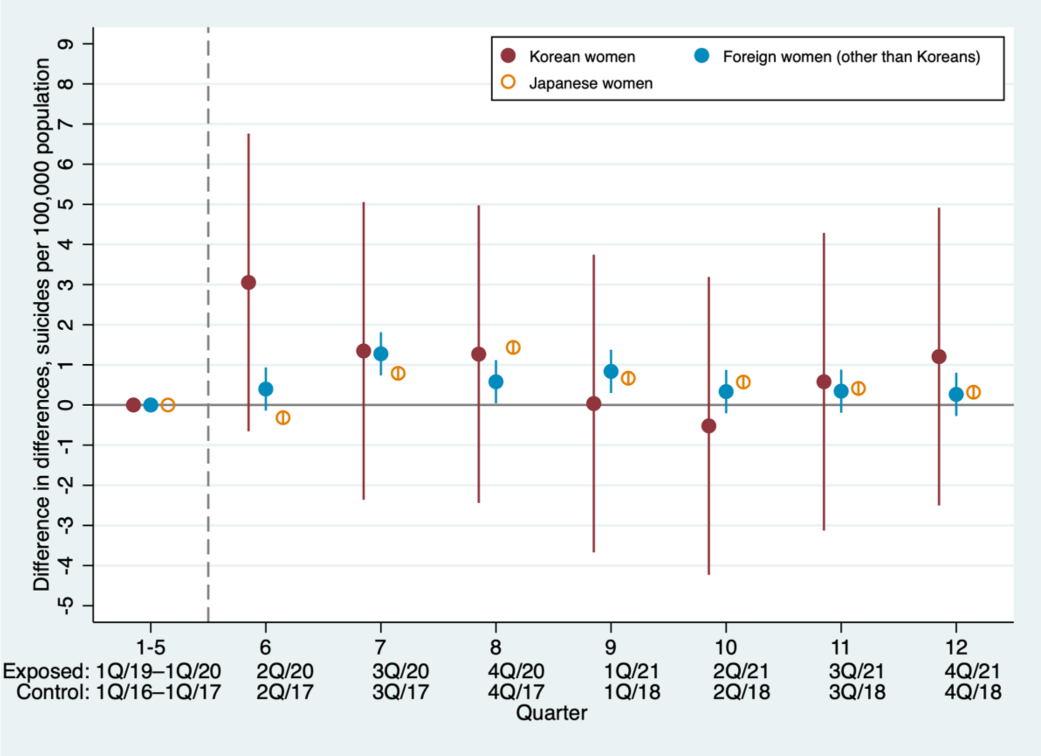

Note: The bars indicate 95% confidence intervals, and the gray dashed lines indicate the beginning of the COVID-19 pandemic (April 2020).

Supplementary Table 2. Difference-in-difference-in-differences (DDD) estimates of the effects of the COVID-19 pandemic on suicide rates in Korean residents and Japanese citizens.

	Men			Women		
	Japanese citizens	Korean residents		Japanese citizens	Korean residents	
	DD estimates (95% CI)	DD estimates (95% CI)	DDD estimates, Korean residents vs. Japanese (95% CI)	DD estimates (95% CI)	DD estimates (95% CI)	DDD estimates, Korean residents vs. Japanese (95% CI)
Q2, 2020	-0.90*** (-1.12, -0.68)	2.39 (-0.19, 4.97)	3.29** (1.36, 5.22)	-0.32** (-0.46, -0.17)	3.05 (-0.66, 6.76)	3.37* (0.60, 6.14)
Q3, 2020	0.18 (-0.04, 0.41)	-2.21 (-4.80, 0.37)	-2.40* (-4.33, -0.47)	0.79*** (0.64, 0.94)	1.35 (-2.36, 5.06)	0.56 (-2.21, 3.32)
Q4, 2020	1.24*** (1.02, 1.46)	6.39** (3.81,8.97)	5.15*** (3.22, 7.08)	1.44*** (1.29, 1.58)	1.27 (-2.44, 4.98)	-0.17 (-2.93, 2.60)
Q1, 2021	0.44** (0.22, 0.66)	1.14 (-1.45, 3.72)	0.70 (-1.23, 2.63)	0.67*** (0.52, 0.81)	0.04 (-3.67, 3.74)	-0.63 (-3.40, 2.14)
Q2, 2021	0.50* (0.28, 0.73)	-1.20 (-3.78, 1.38)	-1.70 (-3.63, 0.23)	0.57*** (0.43, 0.72)	-0.52 (-4.23, 3.19)	-1.09 (-3.86, 1.67)
Q3, 2021	0.17 (-0.05, 0.39)	4.30* (1.72, 6.88)	4.13*** (2.20, 6.06)	0.41** (0.27, 0.56)	0.58 (-3.13, 4.29)	0.17 (-2.60, 2.93)
Q4, 2021	-0.07 (-0.29, 0.16)	4.57** (1.99, 7.15)	4.63*** (2.70, 6.56)	0.32** (0.17, 0.47)	1.21 (-2.50, 4.92)	0.89 (-1.88, 3.65)

Abbreviations: DD, difference-in-differences; DDD, difference-in-difference-in-differences; CI, confidence interval; Q, Quarter

* P<0.05, ** P<0.01, ***P<0.001.
